# Multimodality, interaction modeling, and multimodule architectures in genomic prediction: A unified conceptual framework

**DOI:** 10.1002/tpg2.70278

**Published:** 2026-07-23

**Authors:** J. Crossa, J. Sun, A. Montesinos‐López, P. Pérez‐Rodríguez, P. Vitale, S. Pérez‐Elizalde, R. Howard, O. A. Montesinos López

**Affiliations:** ^1^ Department of Statistics and Data Science Post‐Graduate College (COLPOS) Montecillo México; ^2^ Department of Statistics, School of Science Yanshan University Qinhuangdao China; ^3^ Centro Universitario de Ciencias Exactas e Ingenierías (CUCEI) Universidad de Guadalajara Guadalajara México; ^4^ International Maize and Wheat Improvement Center (CIMMYT) Texcoco México; ^5^ Department of Statistics University of Nebraska–Lincoln Lincoln Nebraska USA; ^6^ Facultad de Telemática Universidad de Colima Colima México

## Abstract

The rapid expansion of genomic, environmental, phenomic, and other high‐dimensional data sources has transformed genomic prediction in plant breeding. However, the terms *multimodal*, *interaction modeling*, and *multimodule architecture* are often used inconsistently, generating ambiguity regarding whether they refer to biological assumptions, data integration strategies, or computational design. These dimensions are conceptually independent in the sense that none logically requires or implies the others. Interaction modeling may be implemented within a multimodule architecture, but modular computation does not inherently encode biological interaction. Multimodality refers strictly to the joint use of heterogeneous biological data sources; interaction modeling reflects explicit assumptions about biological dependencies such as genotype‐by‐environment effects; and multimodularity describes how computation is architecturally organized. We illustrate the proposed framework using conceptual and literature‐based examples from wheat breeding, emphasizing interpretation rather than introducing new experimental results. By clarifying terminology and model design principles, this framework aims to improve methodological transparency, facilitate fair comparison among prediction approaches, and strengthen communication between quantitative geneticists, data scientists, and breeding practitioners. While often grouped under the umbrella of artificial intelligence, the approaches used here are more precisely framed as statistical learning methods designed to model and predict measurable genotype–environment–phenotype relationships rather than to generate synthetic or human‐like outputs.

## Plain Language Summary

Plant breeders use genomic prediction to help decide how well crops like wheat will grow before fully testing them in fields. This helps speed up breeding for better yields, but terms such as “multimodal data” (multi‐type data like markers, weather data, and plant sensor readings), “interaction modeling” (how genes respond differently in various environments), and “multimodule architecture” (splitting computer tasks) are often mixed up. The paper's purpose is to separate these ideas clearly with a new framework, using wheat examples to show they work independently in statistical models or deep learning methods. It shows that multimodality just means using varied data without the assumption of automatic interactions. Interactions need explicit setup (for example, genotype × environment terms), and modules are about computer design, not biology. By clarifying these, the framework improves transparency in breeding research.

**Abbreviations**
AI: artificial intelligence
GBLUP: genomic best linear unbiased prediction
HTP: high‐throughput phenotyping
NDVI: normalized difference vegetation index
RKHS: reproducing kernel Hilbert space
UAV: unmanned aerial vehicle

## 1 INTRODUCTION

Genomic prediction has become a foundational component of modern plant breeding by enabling estimation of breeding values using genome‐wide molecular markers, often prior to extensive phenotypic evaluation (Crossa et al., 2017; Meuwissen et al., 2001; VanRaden, 2008). The development of genomic best linear unbiased prediction (GBLUP) and related linear mixed‐model approaches provided a statistically rigorous framework grounded in quantitative genetic theory, allowing breeders to leverage dense marker information to accelerate selection cycles and increase genetic gain.

As breeding programs increasingly operate across heterogeneous environments, accurate prediction requires explicit consideration of genotype‐by‐environment (G × E) interaction. Classical studies on phenotypic stability laid the foundation for understanding differential genotype responses across environments (Finlay & Wilkinson, 1963; Perkins & Jinks, 1968). These concepts were later formalized within mixed‐model frameworks for multi‐environment trials and extended to genomic‐enabled reaction norm models integrating molecular markers directly into G × E modeling (Burgueño et al., 2012; Jarquín et al., 2014). Semi‐parametric approaches such as reproducing kernel Hilbert space (RKHS) regression further expanded flexibility by capturing nonlinear genetic effects while maintaining statistical interpretability (Crossa et al., 2010; Cuevas et al., 2016; de los Campos et al., 2010).

Parallel advances in statistical modeling have been accompanied by rapid expansion in both the quantity and diversity of data collected. Environmental covariates describing weather, soil, and management practices are now routinely available for multi‐environment trials. High‐throughput phenotyping (HTP) platforms based on proximal sensors and unmanned aerial vehicles (UAVs) provide dynamic measurements of canopy traits and vegetation indices such as normalized difference vegetation index (NDVI) (Araus & Cairns, 2014; Heslot et al., 2014). Transcriptomic, metabolomic, and other omics layers are increasingly incorporated into breeding pipelines.

The integration of these heterogeneous data sources is often described as multimodal genomic prediction. Recent studies show that combining genomic, environmental, and phenomic information can enhance prediction accuracy, particularly under sparse or forward‐prediction scenarios (Crossa et al., 2021; Jarquín et al., 2014; Montesinos‐López et al., 2023).

Deep‐learning approaches expand these possibilities through flexible representation learning (Baião et al., 2025; Ballard et al., 2024; Montesinos‐López et al., 2019; Montesinos‐López, Montesinos‐Lopez, Crossa, et al., 2018; Montesinos‐López et al., 2021; Montesinos‐López et al., 2024). Recent advances in machine learning have led to their widespread association with artificial intelligence (AI) across many disciplines, including agriculture. In crop science and plant breeding, however, these approaches are most appropriately viewed as extensions of statistical learning and quantitative genetics. Rather than emulating cognitive processes, they provide flexible mathematical frameworks for estimating relationships among genomic information, environmental variation, and observed phenotypes.

In the quantitative genetics and applied statistics literature, the use of heterogeneous information sources has sometimes been described as *multi‐type data integration*, whereas the machine learning community more commonly uses the term *multimodal data*. In this paper, we treat these expressions as equivalent when they refer to the joint use of distinct biological data sources. By contrast, the term *multimodule* is typically used in machine‐learning contexts to describe computational architectures composed of separate processing components. These uses are conceptually different, although the terminology is sometimes conflated in literature.

The terms multimodal, interaction‐aware, multimodule, and multimodel are often used interchangeably despite referring to distinct conceptual dimensions. In some cases, inclusion of multiple data types is interpreted as evidence of biological interaction modeling, even when modalities are combined additively. In others, modular neural architectures are assumed to imply interaction‐aware inference, despite the absence of explicit cross‐modality terms. Conversely, linear mixed models incorporating G × E interaction are sometimes not recognized as multimodal when environmental covariates are included.

This conflation complicates model interpretation, obscures biological assumptions, and hampers fair methodological comparison. For breeding programs operating under resource and time constraints, lack of clarity in model specification undermines transparency and reproducibility.

Evidence of such conflation can be found across the genomic prediction literature, where terms such as “multimodal,” “multimodule,” and “interaction‐aware” are used inconsistently or without explicit distinction (e.g., Crossa et al., 2021). Table 1; (Kick et al., 2023; Montesinos‐López et al., 2023) summarizes representative studies and reinterprets them under the framework proposed here, highlighting common sources of ambiguity.

**TABLE 1:** Representative examples of terminology usage in genomic prediction studies and reinterpretation under the proposed framework.

Study	Terminology used by authors	Data modalities (G, E, P, etc.)	Interaction modeling	Multimodule architecture	Interpretation under proposed framework	Source of ambiguity
Montesinos‐López et al. (2023)	“Multimodal deep learning”	G + P + E	Model‐dependent (explicit in LMM, implicit in DL)	Yes (structured DL)	Multimodal + partially interaction‐aware + multimodule	Multimodality often interpreted as interaction modeling
Kick et al. (2023)	“Integrated deep learning model”	G + E + management	Implicit (through features)	No explicit modular structure	Multimodal + interaction‐aware (implicit) + single‐module	Multimodality conflated with modularity
Crossa et al. (2021)	“Genomics–phenomics–enviromics integration”	G + P + E	Limited or not explicit	No	Multimodal + largely additive	Multimodal interpreted as biologically interactive
Jarquín et al. (2014)	“Reaction norm model”	G + E	Yes (explicit G × E)	No	Interaction‐aware + not multimodule	Interaction not recognized as multimodal structure
Cuevas et al. (2016)	“Kernel G × E model”	G + E	Yes	No	Interaction‐aware + nonlinear + non‐modular	Nonlinearity conflated with multimodality
Heslot et al. (2014)	“Environmental covariate integration”	G + E	Partial (model‐dependent)	No	Multimodal + partially interaction‐aware	Additive integration interpreted as interaction
Montesinos‐López, Montesinos‐López, et al. (2018)	“Deep learning genomic prediction”	G	Implicit nonlinear	Yes (neural network)	Unimodal + nonlinear + multimodule	Multimodule conflated with multimodal
de los Campos et al. (2010)	RKHS genomic prediction	G	Implicit nonlinear	No	Unimodal + nonlinear	Nonlinearity interpreted as interaction
Burgueño et al. (2012)	“G × E mixed model”	G + E	Yes (explicit covariance structure)	No	Interaction‐aware + not multimodule	Classical interaction not framed as multimodal
Crossa et al. (2010)	Genomic prediction	G	No	No	Unimodal + additive	Baseline model, no ambiguity

Abbreviations: DL, deep learning; LMM, linear mixed model; RKHS, reproducing kernel Hilbert space.

The examples summarized in Table 1 illustrate that the same modeling approaches may be described using different terminology, and conversely, similar terms may refer to conceptually distinct model properties. This inconsistency makes it difficult to interpret model assumptions, compare methodologies, and assess the biological meaning of reported results. In particular, the use of multiple data sources is often conflated with interaction modeling, and the adoption of modular computational structures is sometimes interpreted as evidence of biologically meaningful dependencies. These observations motivate the need for a clear conceptual framework that separates data integration, biological assumptions, and computational design into distinct and explicitly defined dimensions.

We propose a unified framework that disentangles three independent dimensions of genomic prediction design: (1) multimodal data integration (what information is used), (2) biological interaction modeling (what dependencies are assumed), and (3) multimodule computational architecture (how computation is organized). These dimensions define distinct aspects of model design and can be combined flexibly across modeling approaches. Clarifying these distinctions supports transparent comparison and context‐aware model selection in modern breeding programs.

## 2 CONCEPTUAL FRAMEWORK: MULTIMODALITY, INTERACTION, AND MODULARITY

The rapid expansion of heterogeneous data sources and computational approaches has increased both the power and the conceptual complexity of genomic prediction models. To improve clarity and comparability across studies, it is essential to distinguish among three independent but frequently conflated dimensions of model design: (i) *multimodal data integration* (what biological information is used), (ii) *biological interaction modeling* (what dependency assumptions are encoded), and (iii) *multimodule computational architecture* (how computation is organized).

A formal mathematical justification of these conceptual distinctions is provided in the Appendix, demonstrating that multimodality, interaction modeling, and multimodule architecture are mathematically independent properties.

**FIGURE 1:**
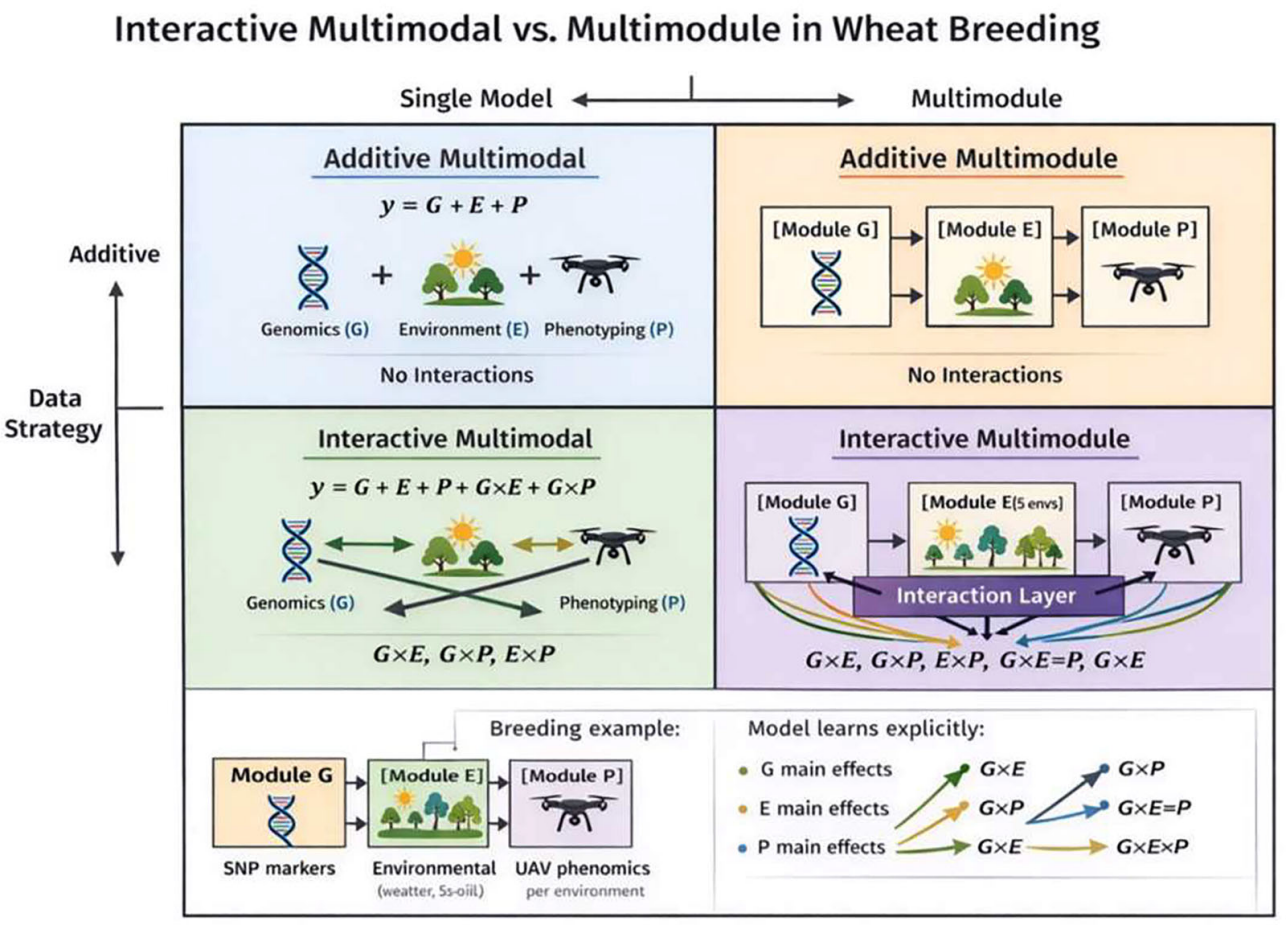
Provides a schematic guide to the conceptual framework adopted in this study. The left part of the figure illustrates multimodal data integration, where multiple biological information sources—genomic markers (G), environmental covariates (E), and phenotypic or phenomic descriptors (P)—are jointly used as model inputs. This representation emphasizes that multimodality is a property of the data and does not, by itself, imply any assumption about biological interactions among data sources. For clarity, all schematic model representations implicitly include a residual error term (ε), representing unexplained variation and measurement noise, although it is not shown explicitly in the left panels to preserve visual simplicity.

Figure 1 provides a schematic overview of these dimensions. The left panel illustrates multimodal data integration, while the right panel represents multimodule computational organization. Interaction modeling operates conceptually across modalities and is independent of architectural design. Formal mathematical support for these distinctions is provided in the Appendix.

The right panel of Figure 1 illustrates multimodule model architectures, in which the modeling process is decomposed into separate computational components that are later integrated to generate predictions. Each module is represented schematically as an independent processing unit, emphasizing the architectural separation of model components rather than any specific biological assumption. This visual representation highlights that multimodule design is a computational choice, complementary to but distinct from the use of multimodal data.

### 2.1 Multimodal data: Biological information sources

We begin by clarifying multimodality as a property of the data inputs, independent of model structure or biological assumptions.

Multimodal data integration refers strictly to the joint use of multiple biological information sources within a prediction model. In plant breeding, the most common modalities include *genomic data (G)*: genome‐wide molecular markers such as single‐nucleotide polymorphisms; *environmental covariates (E)*: weather summaries, soil properties, management descriptors, and derived environmental indices (enviromics); and *phenomic traits (P)*: HTP measurements, including vegetation indices (e.g., NDVI), canopy temperature, and spectral reflectance.

Additional modalities may include transcriptomics, metabolomics, ionomics, or management variables, depending on the breeding context. Under this definition, multimodality is a property of the *inputs* to the model. A model is multimodal if it uses two or more distinct biological data types, regardless of how they are combined or processed.

Importantly, multimodality does not imply interaction modeling. Multiple modalities may be incorporated additively, where each data source contributes independently to prediction. Improved predictive performance in such models indicates complementary information across modalities but does not, by itself, demonstrate biological dependence among them.

In practice, multimodal data integration often requires careful preprocessing due to differences in dimensionality, temporal resolution, and measurement scale. For example, genomic markers are high‐dimensional and static across environments, whereas environmental covariates are environment‐specific and often temporally structured. HTP traits may vary dynamically during the growing season. Harmonizing these inputs addresses technical compatibility but does not constitute interaction modeling.

While multimodality concerns the types of data included in a model, it does not specify how these data sources relate to each other biologically. This distinction leads naturally to the concept of interaction modeling, which addresses dependencies among modalities.

### 2.2 Interaction modeling as a biological assumption

Interaction modeling represents a distinct conceptual dimension, focusing on whether and how dependencies among data modalities are explicitly encoded within the model. Interaction modeling reflects explicit biological hypotheses about dependencies among data modalities. The most prominent example in plant breeding is G × E interaction, where genotypic performance varies systematically across environmental conditions.

In statistical terms, interaction effects represent cross‐modality dependencies that cannot be expressed as independent additive contributions. In biological terms, they capture differential adaptation, environmental sensitivity, and context‐specific expression of genetic potential.

Crucially, interaction modeling is independent of computational architecture. A formal demonstration of this independence is provided in the Appendix. Genotype‐by‐environment interaction can be encoded within linear mixed models and reaction norm formulations, kernel‐based approaches such as RKHS, machine learning algorithms, and deep learning architectures.

Nonlinear models may capture complex statistical patterns; however, this does not imply that biologically meaningful cross‐modality interactions are explicitly represented. Conversely, classical linear mixed models can incorporate such interactions explicitly through covariance structures.

Having distinguished interaction modeling as a biological assumption, we next consider multimodule architectures, which concern the computational organization of prediction models rather than their biological interpretation.

### 2.3 Multimodule models as computational architecture

Multimodule architectures define how computation is structured within a model, independently of the data modalities used or the biological assumptions encoded. Multimodule models refer to computational architectures in which prediction is decomposed into separate components or modules. This concept is architectural rather than biological. In multimodule designs, different modules may process subsets of inputs independently before integration. For example, one module may process genomic markers, another environmental covariates, and a third phenomic traits. Their outputs are later combined to generate predictions.

However, multimodularity does not necessarily align with data modality. Modules may be defined for computational convenience, hierarchical representation learning, or regularization, without explicit biological interpretation.

Importantly: A multimodal model may be implemented as a single unified computational architecture; a multimodule model may operate on a single data modality. Thus, multimodularity concerns **how computation is organized**, not what information is used or what biological dependencies are assumed.

### 2.4 Generic versus structured multimodule architectures

The conceptual distinctions developed in this study have direct implications for model design, interpretation, and deployment in genomic prediction. As illustrated in Table 1, conflation of multimodality, interaction modeling, and multimodule architecture can lead to ambiguity in how models are interpreted and compared. In practical terms, this may result in incorrect assumptions about biological mechanisms, overestimation of model capabilities, and reduced reproducibility across studies.

**Generic multimodule architectures** define modules primarily to enhance computational flexibility or representation capacity. After transformation, modality identity may be lost in a shared latent representation. While such designs may achieve high predictive accuracy, interpretability can be limited.

**Structured multimodule architectures**, in contrast, explicitly align modules with biological modalities. Separate encoders are assigned to genomic, environmental, phenomic, or other data sources, preserving their identity throughout the modeling process. Integration occurs at a defined fusion stage.

Structured architectures may facilitate: Interpretability, transparent reporting, modular updating of components, and alignment with breeding decision processes.

In operational breeding contexts—where decisions must be justified and models deployed across years—such interpretability can be as important as predictive performance.

For example, a generic multimodule architecture may consist of multiple neural subnetworks whose outputs are merged into a shared latent representation, without preserving the identity of individual data modalities. In contrast, a structured multimodule architecture assigns dedicated processing components to genomic, environmental, and phenomic inputs, maintaining their separation throughout the modeling process and enabling clearer interpretation of their contributions.

The choice between generic and structured multimodularity should therefore reflect breeding objectives, data availability, and interpretability requirements rather than perceived methodological sophistication.

Together, these three dimensions—multimodality, interaction modeling, and multimodularity—define a unified conceptual space for describing genomic prediction models. Explicitly distinguishing among them provides a foundation for clearer model specification, interpretation, and comparison, which is further illustrated through practical examples in the following section.

### 2.5 Multimodel analyses and model comparison

A separate but related concept is multimodel analysis, which refers to the evaluation and comparison of multiple distinct modeling frameworks (e.g., GBLUP, RKHS, gradient boosting, and deep learning).

Multimodel comparison is an analytical strategy aimed at assessing robustness and performance across approaches. It is independent of whether individual models are multimodal, interaction‐aware, or multimodule. To improve transparency and reproducibility, genomic prediction studies should explicitly report: Which data modalities are included? Whether biological interactions are modeled and how; Whether modular architecture is used and how modules align with data sources; the rationale for comparing alternative models. Making these dimensions explicit strengthens methodological clarity and facilitates meaningful comparison across studies.

Because terminology varies across disciplines, the same concepts are often described using different expressions in quantitative genetics, statistics, and machine‐learning literature. Table 2 summarizes common terms found in the literature and clarifies how they are used and distinguished in this study.

**TABLE 2:** Terminology used across disciplines and definitions adopted in this study.

Term used in literature	Typical field of use	Meaning in original context	Definition adopted in this paper
Multimodal data	Machine learning, AI	Multiple input data sources	Joint use of heterogeneous biological data (G, E, P, omics)
Multi‐type data	Statistics, quantitative genetics	Different classes of predictors	Equivalent to multimodal data integration
Multimodule architecture	Deep learning	Separate subnetworks or processing blocks	Computational organization of a model, independent of biological assumptions
Multimodal network (ambiguous usage)	Some DL literature	Often refers to modular architectures	Distinguished here from multimodal data; refers to architecture, not data
Multimodel	Statistical modeling, prediction studies	Comparing different models	Analytical strategy for evaluation, not a model property

## 3 PRACTICAL USES IN GENOMIC PREDICTION STUDIES

The conceptual distinctions introduced above are not merely theoretical. They directly influence how genomic prediction models are specified, interpreted, and compared in practical breeding contexts. To illustrate this, consider a wheat breeding program evaluating a set of cultivars across multiple site–year combinations. For each cultivar–environment combination, breeders may have access to genome‐wide molecular markers (G), environmental covariates describing weather and soil conditions (E), HTP traits derived from UAV platforms (P), and potentially additional omics layers measured in subsets of environments.

Within this setting, several modeling choices arise: (1) Whether to include one or multiple data modalities (multimodality); (2) Whether to encode explicit genotype‐by‐environment or cross‐modality interactions (interaction modeling); (3) Whether to implement the model as a unified computational structure or as a modular architecture (multimodularity). These choices are independent and can be combined flexibly.

### 3.1 Structured multimodal and multimodule example: Montesinos‐López et al. (2023)

Montesinos‐López et al. (2023) provide a clear example of multimodal genomic prediction in wheat breeding. In their study, genomic markers, UAV‐derived NDVI traits, and environmental indicators were integrated within multiple prediction frameworks.

From the perspective of this framework, (1) the study is **multimodal**, as it jointly uses genomic and phenomic data. (2) Interaction modeling depends on the specific implementation. In linear mixed‐model formulations, genotype‐by‐environment interaction can be explicitly parameterized. (3) The deep‐learning implementation adopts a **structured multimodule architecture**, with separate subnetworks assigned to different data modalities before integration.

Importantly, the presence of multimodal data does not automatically imply that biological interactions are explicitly modeled. Likewise, the use of a multimodule neural architecture does not inherently encode interpretable genotype‐by‐environment effects unless explicitly structured to do so. This example demonstrates how multimodality, interaction modeling, and modularity can coexist within the same study while remaining conceptually distinct.

### 3.2 Multimodal data integration without explicit multimodule architecture: Kick et al. (2023)

Kick et al. (2023) investigated yield prediction through integration of genetic, environmental, and management data. Their analysis is unambiguously multimodal, as it combines multiple sources of biological and contextual information.

However, the models are implemented within unified computational frameworks rather than explicitly structured multimodule architectures. Interaction effects are incorporated through model specification and feature representation rather than through modular decomposition aligned with biological data types.

This example reinforces a central message of this study: multimodal data integration and interaction modeling can be achieved without adopting a multimodule architecture. Conversely, multimodule architectures are not required for multimodal prediction.

**FIGURE 2:**
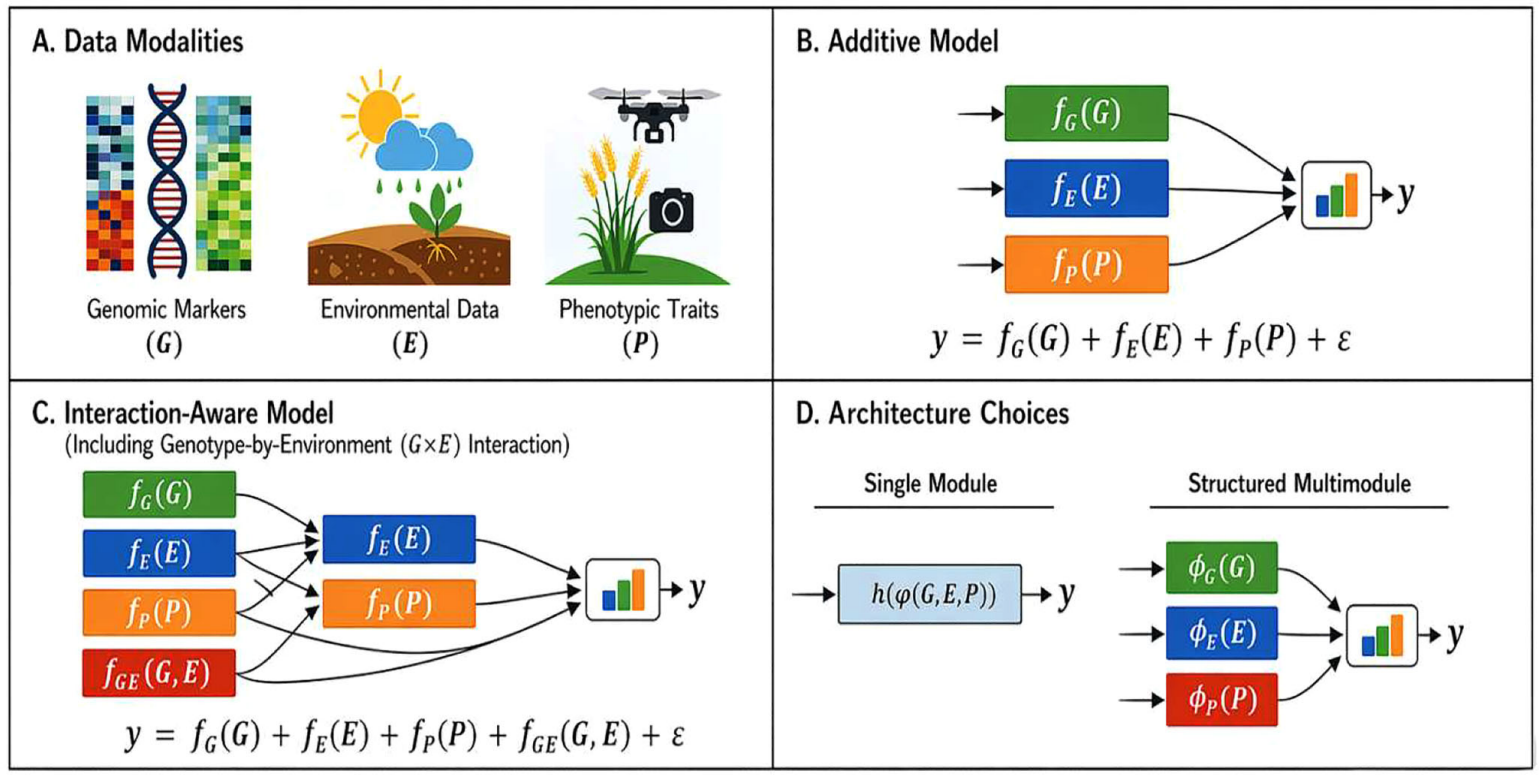
Integrated representation of model design dimensions.

Figure 2 synthesizes the framework by jointly illustrating the following (1) Multimodal data integration (what information is used), (2) Interaction‐aware modeling (what biological assumptions are encoded), (3) Alternative architectural implementations (single‐module versus structured multimodule). While Figure 1 presents the conceptual separation of these dimensions, Figure 2 illustrates their joint implementation within practical modeling scenarios.

The Figure 2 emphasizes that these dimensions represent complementary components of model design that may be combined in different ways.

With these practical illustrations, we now turn to the broader implications of this framework for genomic prediction design, reporting practices, and breeding decision‐making.

## 4 IMPLICATIONS FOR GENOMIC PREDICTION AND BREEDING PROGRAMS

As illustrated in Table 1, conflation of multimodality, interaction modeling, and multimodule architecture has direct implications for how models are interpreted, compared, and deployed in breeding programs. These conceptual distinctions are formally supported in the Appendix.

### 4.1 Model design and biological interpretation

Multimodal data integration is an information design choice. Including genomic, environmental, phenomic, or management data does not imply interaction modeling. Interaction effects—such as G × E—must be explicitly encoded through covariance structures or cross‐modality terms.

Whether cross‐modality interactions are captured depends on model specification. Classical mixed models or kernel formulations incorporate interaction through explicitly defined terms (e.g., genotype × environment covariance structures). Deep‐learning models with hidden layers may also represent complex nonlinear dependencies among inputs; however, these interactions are learned implicitly through latent representations and are typically not parameterized in a way that allows direct biological interpretation.

### 4.2 Interaction modeling and biological insight

For breeding programs targeting adaptation across heterogeneous environments, explicit modeling of G × E interaction may be critical for reliable forward prediction. Interaction modeling is independent of algorithmic class and can be implemented in linear mixed models, kernel methods, or deep‐learning frameworks. A formal demonstration of this independence is provided in the Appendix.

Importantly, the presence of nonlinear modeling does not guarantee that biologically meaningful interactions are captured. Without explicit cross‐modality structure, even complex models may remain effectively additive from a biological perspective. This distinction is critical when interpreting model outputs in breeding contexts.

### 4.3 Implications for breeding decision‐making

In practical breeding programs, predictive accuracy is only one component of model utility. Robustness across years, interpretability of results, data availability, and ease of deployment are equally important considerations. Conflating multimodality, interaction modeling, and multimodule architecture may lead breeders to select models based on perceived methodological sophistication rather than alignment with biological objectives.

For example, a multimodal model that integrates genomic and environmental data additively may provide stable and interpretable predictions without requiring complex architecture. Conversely, when predicting performance in new or highly variable environments, explicit modeling of genotype‐by‐environment interaction may be essential. In such cases, the choice of model should prioritize biological relevance rather than computational complexity.

Structured multimodule architecture may be advantageous when dealing with heterogeneous or partially observed data sources, particularly in large‐scale breeding programs. However, their use should be justified in terms of data structure and operational needs rather than assumed superiority.

These considerations highlight the importance of explicitly distinguishing among data integration, interaction modeling, and computational design when selecting and evaluating genomic prediction models.

### 4.4 Reporting and design guidelines for genomic prediction studies

To improve transparency and reproducibility, we propose that genomic prediction studies explicitly report three independent dimensions of model design:

(i) multimodal data integration: which biological data sources are used (e.g., genomic, environmental, phenomic, and omics);
(ii) interaction modeling: whether cross‐modality dependencies such as genotype‐by‐environment interactions are explicitly modeled and how they are encoded;
(iii) multimodule architecture: whether computation is organized into separate modules and whether these modules correspond to biological data sources.

Explicit reporting of these elements would facilitate fair comparison among models, improve interpretability, and support informed decision‐making in breeding programs. Adoption of such reporting standards would also reduce ambiguity in terminology and strengthen communication across disciplines.

## 5 WHY DEEP LEARNING FOR MULTIMODAL DATA INTEGRATION?

Integrating genomic, environmental, phenomic, and omics data presents methodological challenges due to differences in dimensionality, structure, and temporal resolution.

Linear mixed models such as GBLUP provide robustness and interpretability but rely primarily on additive covariance structures. Kernel methods, including RKHS, extend flexibility by allowing nonlinear similarity patterns while preserving statistical grounding. Classical machine‐learning algorithms offer additional nonlinear capacity but often require careful feature engineering to integrate heterogeneous inputs.

Deep‐learning architectures use representation learning to extract latent embeddings from high‐dimensional and structured inputs. Structured multimodule designs may assign modality‐specific encoders and fuse learned representations for prediction. This flexibility can be advantageous when large datasets are available.

However, deep learning does not inherently encode biologically interpretable interaction effects unless explicitly structured to do so. Performance gains depend strongly on data volume, regularization, and validation design. Increased complexity may reduce interpretability and complicate deployment.

Deep learning should therefore be viewed as a complementary tool rather than a universal replacement for established statistical approaches. Recognizing this distinction helps place machine learning approaches within the established scientific paradigm of crop improvement, where advances are measured by their ability to better represent biological complexity and support selection decisions.

## 6 CONCLUSIONS AND OUTLOOK

The integration of genomics, enviromics, phenomics, and omics technologies has increased both the opportunities and conceptual complexity of genomic prediction. As methods diversify, clarity in terminology and model specification becomes essential.

This study disentangles three independent dimensions of model design:

- multimodal data integration (what information is used),
- biological interaction modeling (what dependencies are assumed),
- multimodule computational architecture (how computation is organized).

Recognizing these distinctions improves clarity in model specification, interpretation, and comparison across genomic prediction studies. Future methodological development should report and evaluate these dimensions independently rather than equating architectural novelty with biological insight. Explicit articulation of data integration strategies, interaction assumptions, and computational design choices will strengthen reproducibility, interpretability, and responsible deployment of prediction systems.

As breeding programs move toward increasingly data‐rich environments, principled frameworks for describing and comparing modeling strategies will be critical for ensuring that methodological innovation translates into biological understanding and sustainable genetic gain.

Appendix is intended to provide formal support for the conceptual claims made in the main text, rather than to introduce new methodological developments.

## AUTHOR CONTRIBUTIONS

**J. Crossa**: Investigation; methodology; writing—original draft; writing—review and editing. **J. Sun**: Methodology; writing—original draft; writing—review and editing. **A. Montesinos‐López**: Methodology; writing—original draft; writing—review and editing. **P. Pérez‐Rodríguez**: Methodology; writing—original draft; writing—review and editing. **P. Vitale**: Writing—review and editing. **S. Pérez‐Elizalde**: Writing—review and editing. **R. Howard**: Funding acquisition; methodology; writing—original draft; writing—review and editing. **O. A. Montesinos López**: Methodology; writing—original draft; writing—review and editing.

## CONFLICT OF INTEREST STATEMENT

The authors declare no conflicts of interest.

## FORMAL SUPPORT FOR THE CONCEPTUAL FRAMEWORK

This appendix provides a formal representation of the conceptual distinctions developed in the main manuscript. The objective is not to introduce new methodological developments, but to demonstrate mathematically that:

- Multimodality does not imply interaction modeling.
- Interaction modeling is independent of computational architecture.
- Multimodularity is an architectural property, not a biological one.

Throughout this appendix, bold lowercase letters denote vectors (e.g., 𝐲, 𝐮, and 𝐡). Bold uppercase letters denote matrices (e.g., 𝐆, 𝐄, 𝐗, 𝐙, and 𝐊). Regular font denotes scalars (e.g., μ, σ^2^, and β).

### 1 General prediction setting

Let 𝐲 denote the vector of observed phenotypic responses for n genotype–environment combinations.

$$y\;\in \;R^{n}$$

Let 𝐆 be the genomic marker matrix, where p_G is the number of markers.

$$G\;\in \;{R^{n\times p}}_{G}$$

Let 𝐄 be the environmental covariates matrix, where p_E is the number of environmental descriptors.

$$E\;\in \;{R^{n\times p}}_{E}$$

Let 𝐏 be the phenomic trait matrix, where p_P is the number of high‐throughput phenotyping variables.

$$P\;\in \;{R^{n\times p}}_{P}$$

A general prediction model can be written as:

$$\hat{y}=\;f\left(G,\;E,\;P\right)$$

where ŷ denotes predicted responses. This formulation defines multimodality at the level of inputs. The function f(·) may represent a linear mixed model, a kernel method, or a neural network. Importantly, at this stage, no assumption about interaction has been made.

### 2 Multimodal does not imply interaction

A purely additive multimodal model can be expressed as follows:

$$y=i\mu +f_{G}\left(G\right)+f_{E}\left(E\right)+f_{P}\left(P\right)+e$$

where 𝐢 is a vector of ones, μ is the overall mean, f_G(𝐆) represents genomic effects, f_E(𝐄) represents environmental effects, f_P(𝐏) represents phenomic effects, and 𝐞 is the residual vector.

Assume:

$$e\;∼\;N\left(0,\;\sigma^{2}I\right)$$

In this additive formulation, each modality contributes independently to prediction. There is no term linking genomic and environmental effects.

Formally, this means:

$$f_{GE}\left(G,\;E\right)=0$$

Thus, the model is fully multimodal but contains no biological interaction. This demonstrates that multimodality does not imply interaction modeling.

### 3 Explicit interaction modeling

To encode genotype‐by‐environment interaction, the model becomes:

$$y=i\mu +f_{G}\left(G\right)+f_{E}\left(E\right)+f_{GE}\left(G,\;E\right)+e$$

The function f_GE(𝐆, 𝐄) represents cross‐modality dependence. This term cannot be decomposed as f_GE(𝐆, 𝐄) ≠ f_G(𝐆) + f_E(𝐄). Instead, it captures the biological idea that the genomic effect depends on environmental context.

Crucially, this interaction component can be implemented in linear mixed models (through covariance structures), kernel regression, and neural networks. Therefore, interaction modeling is independent of computational architecture.

### 4 Multimodule architecture without interaction

Consider a generic multimodule neural architecture:

$$h=\varphi \left(G,\;E,\;P\right)$$

$$\hat{y}=\psi \left(h\right)$$

where φ(·) maps inputs into a latent representation space, 𝐡 is a vector of learned features, and ψ(·) maps latent features to predicted outcomes.

Even if computation is modular, the model remains biologically additive if:

$$\psi \left(\varphi \left(G,\;E,\;P\right)\right)=f_{G}\left(G\right)+f_{E}\left(E\right)+f_{P}\left(P\right)$$

Thus, multimodularity does not imply biological interaction.

### 5 Structured multimodule representation

In a structured multimodule architecture:

$$h_{G}=\varphi_{G}\left(G\right)$$

$$h_{E}=\varphi_{E}\left(E\right)$$

$$h_{P}=\varphi_{P}\left(P\right)$$

These are fused:

$$h=F\left(h_{G},\;h_{E},\;h_{P}\right)$$

Prediction is then:

$$\hat{y}=\psi \left(h\right)$$

Interaction is biologically encoded only if the fusion function 𝓕 explicitly models cross‐modality dependence (for example, multiplicative or nonlinear cross‐effects). If 𝓕 is purely additive, then the architecture remains additive in biological interpretation. This demonstrates that multimodularity and interaction modeling are distinct properties.

### 6 Linear mixed model as proof of concept

A classical linear mixed model with genomic and interaction components can be written as follows:

$$y=X\beta +Z_{G}\;u_{G}+Z_{GE}\;u_{GE}+e$$

where 𝐗 is the fixed‐effects design matrix, β is a vector of fixed‐effect coefficients, 𝐙_G and 𝐙_GE are incidence matrices, 𝐮_G is the genomic random‐effect vector, 𝐮_GE is the genotype‐by‐environment interaction vector, and 𝐞 is the residual vector.

Assume:

$$u_{G}\;∼\;N\left(0,\;{\sigma_{G}}^{2}K\right)$$

$$u_{GE}\;∼\;N\left(0,\;{\sigma_{GE}}^{2}K_{GE}\right)$$

This formulation shows that genotype‐by‐environment interaction can be explicitly modeled within classical mixed‐model frameworks. Thus, interaction modeling does not require multimodule neural architectures. Multimodularity is not equivalent to biological interaction. Multimodality does not imply cross‐modality dependence.
